# Design and Development of a 5-Channel Arduino-Based Data Acquisition System (ABDAS) for Experimental Aerodynamics Research

**DOI:** 10.3390/s18072382

**Published:** 2018-07-22

**Authors:** Antonio Vidal-Pardo, Santiago Pindado

**Affiliations:** 1 Instituto Universitario de Microgravedad “Ignacio Da Riva” (IDR/UPM), ETSI Aeronáutica y del Espacio, Universidad Politécnica de Madrid, Pza. del Cardenal Cisneros 3, 28040 Madrid, Spain; santiago.pindado@upm.es; 2 Departamento de Sistemas Aeroespaciales, Transporte Aéreo y Aeropuertos (SATAA), ETSI Aeronáutica y del Espacio, Universidad Politécnica de Madrid, Pza. del Cardenal Cisneros 3, 28040 Madrid, Spain

**Keywords:** Arduino, voltmeter, acquisition system, educational technology, low cost

## Abstract

In this work, a new and low-cost Arduino-Based Data Acquisition System (ABDAS) for use in an aerodynamics lab is developed. Its design is simple and reliable. The accuracy of the system has been checked by being directly compared with a commercial and high accuracy level hardware from National Instruments. Furthermore, ABDAS has been compared to the accredited calibration system in the IDR/UPM Institute, its measurements during this testing campaign being used to analyzed two different cup anemometer frequency determination procedures: counting pulses and the Fourier transform. The results indicate a more accurate transfer function of the cup anemometers when counting pulses procedure is used.

## 1. Introduction

Measuring voltage signals at frequencies varying from 1 Hz to 0.5–1 kHz is, in general, compulsory in every scientific lab. Bearing in mind that almost every physical variable can be translated into a voltage signal, there will always be a constant demand for such measurements in these facilities.

The common work at the IDR/UPM Institute, related to experimental aerodynamics [1,2,3,4,5,6,7,8,9,10], cup anemometer calibration and behavior characterization [11,12,13,14,15,16,17,18,19,20,21,22], space components analysis (batteries, solar panels, control systems) [23,24,25,26,27,28], and space thermal analysis [29,30,31,32], has driven the development of the 5-channel Arduino-Based Data Acquisition System (ABDAS) described in the present paper. The purpose of this development is to have a simple but accurate multi-purpose 10–500 Hz sampling rate voltage-data acquisition system capable of being used in different measurement problems from measuring temperatures with thermocouples to wind-tunnel pitot-tube pressure signals.

Different projects in different engineering disciplines and research [33,34,35,36,37,38], have shown the need for low-cost but accurate data acquisition systems in recent years, from electrical engineering [39,40], to quality assessment [41], photovoltaic performance assessment [42,43,44] and renewable energy [45]. It could be also said that these low-cost measurement instruments seems to be increasingly used within the biomedical sector [46,47,48,49,50], and small-satellite space missions [51,52,53,54,55].

Additionally, another important factor to be taken into account is the increase in the use of Arduino boards within engineering academic programs at universities, as it is a quite good methodology to train students in measurement procedures with low-cost but accurate lab-kits [56,57,58,59,60,61,62,63,64,65,66,67]. In the present paper, the design, development and test verification of a new Arduino-Based Data Acquisition System (ABDAS) for its use in an experimental aerodynamics research facility is described. In addition, other possible uses as instrumentation for lab testing students’ training at different subjects (power systems, vibration analysis, thermal control …) from the Master in Space Systems, are considered [28,68,69].

The principal motivation for this work was to develop a low-cost but accurate alternative to the National Instruments NI USB-6210 Data Acquisition System (NIDAS). This accurate and reliable hardware, controlled by LabVIEW^®^ software, is commonly used at IDR/UPM in research related to cup anemometer performance and other wind speed sensors research.

The present paper is organized as follows: the data acquisition system (design, data processing, calibration …) is described in Section 2. The experimental set-up used for its validation is outlined in Section 3, whereas the results are included and discussed in Section 4. Finally, conclusions are summarized in Section 5.

## 2. System Requirements and Design

The design of the Arduino-Based Data Acquisition System (ABDAS) described in the present work has been carried out under the following requirements:

- Low-cost. The data acquisition system is designed for academic purposes, especially for its use in engineering university degrees. The reduced cost of the parts that compose the system makes it affordable for any student (or institution).
- Open-source software. Bearing in mind that the use of this system should fit many different testing experiments, the open-source Arduino software (IDE) was selected in order to allow the users maximum flexibility to program tools for any specific set-up.
- Development based on basic knowledge. The theoretical design of the acquisition system has been based on basic electrical/electronic engineering know-how, as its design should be modified in future and improved versions by Bachelor and Master’s students. High-level elements, electronic designs or concepts have been avoided.
- User-friendly design. The interface with the user at both levels, hardware and software, has been designed as much intuitive as possible in order to ease the initial experiences with the system.
- Finally, the following technical requirements were stated, as the main purpose of this acquisition system is to be use in a research center such as the IDR/UPM Institute:
  ○ 500 Hz sampling rate (at least).
  ○ Five measurement channels (as other physical variables such as temperature, dynamic pressure, humidity, etc. should be measured at the same time as the main variable, which is normally the static pressure or some force).
  ○ Minimum measuring ranges from 0 V to 6 V (analog input with respect to ground).
  ○ 10 mV minimum accuracy along all measuring range.

**Figure 1:**
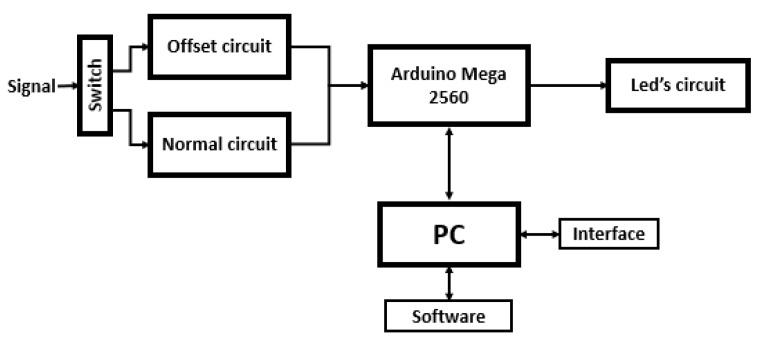
Diagram of the Arduino-Based Data Acquisition System (ABDAS) described in the present work.

Following the above requirements, ABDAS has been developed. A diagram of this system’s design is included in Figure 1. It can be observed in the figure that the core of the system is the Arduino board, its purposed being to acquire and process the data. More specifically, Arduino Mega 2560 was selected as it is an open-source product, inexpensive and provides sufficient analog pins for its possible different future uses at IDR/UPM Institute. In Table 1, the general specifications of this board are included.

**Figure 2:**
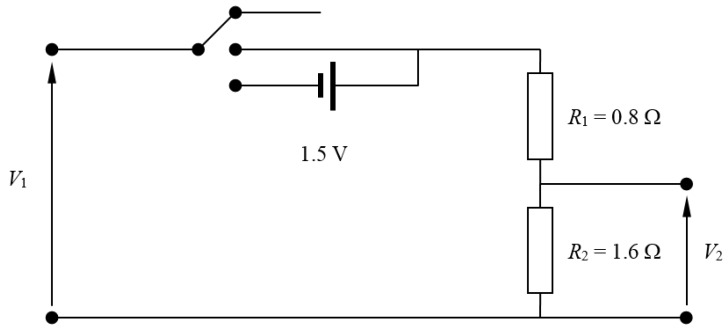
Sketch of the voltage dividers used to enlarge the measuring rang of ABDAS.

It should be underlined that the analog pins of the Arduino board are 10-bit resolution, with a measuring range from 0 V to 5 V. This 10-bit resolution allows us to reach ±4.88 mV accuracy (beyond the technical requirement). With regard to the measuring range and to fulfill the correspondent technical requirement, it was decided to enlarge it by using voltage dividers (see Figure 2) that provide the following ratio between the input signal voltage, *V_in_*, and the output signal voltage, *V_out_*:

(1) $$V_{out}=\frac{R_{2}}{R_{1}+R_{2}}V_{in}=\frac{2}{3}V_{in}$$

where *R*_1_ = 800 mΩ, and *R*_2_ = 1600 mΩ.

**Figure 3:**
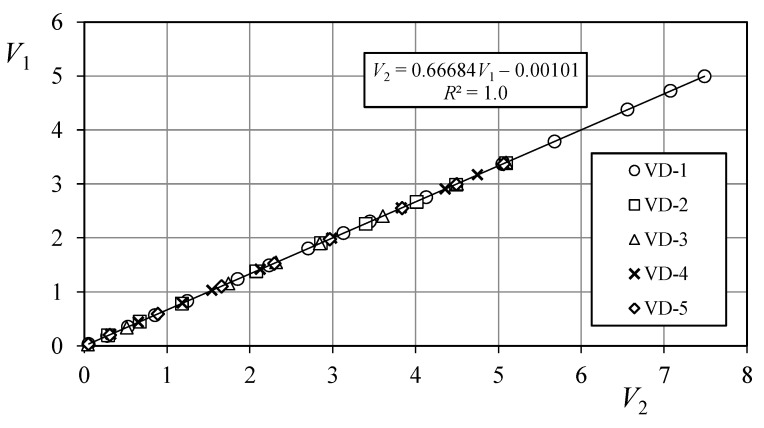
Transfer function (Equation (2)) of ABDAS voltage dividers. The Equation (linear fitting) correspondent to Channel-1 voltage divider (VD-1) is included in the Figure. See also Table 2.

ABDAS is composed by two different electrical circuits. The first one modifies the input signal whereas the second one is a light-signaling circuit. The input signal circuit modifies the signal by using the aforementioned voltage dividers that enlarge the input range to 7.5 V. Therefore, taking into account the 10-bit resolution of the board the accuracy is improved to ±7 mV. It should be also mentioned that the voltage dividers could introduce certain level of error in the measurements. A calibration of each one was carried out. In Figure 3, the results of the measurements obtained are shown. Based on these results a linear transfer function was obtained for each voltage divider:

(2) $$V_{2}=aV_{1}+b$$

where *V*_1_ is the input signal to the voltage divider, and *V*_2_ is the output signal. In Table 2, the coefficients related to each one of the transfer functions are included together with the coefficient of determination, *R*^2^, related to the fittings, and the maximum and average errors of these transfer functions in relation to the measured data. Additionally, a 1.5 V offset is possible to be introduced in each channel of the ABDAS in order to allow the measurement of negative values of the signal (see Figure 2). Therefore, the measurement range can be either [0, 7.5] or [−1.5, 6].

**Figure 4:**
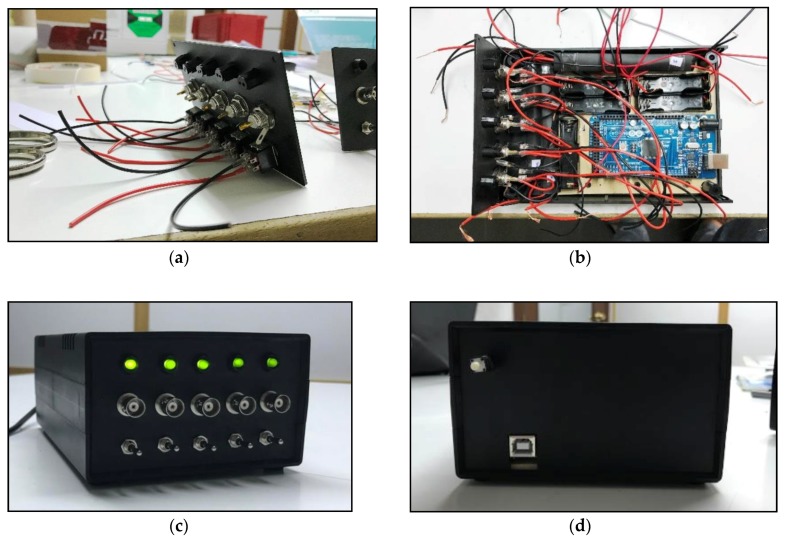
Pictures of ABDAS along its development/manufacturing process. (**a**) Connectors, lightning and switching panel. (**b**) ABDAS distribution inside the enclosure. (**c**) Front view. (**d**) Rear view.

In Figure 4 some pictures describing ABDAS during its integration are included. Each measurement channel is activated by a 3-position switch located at the front panel of the system’s enclosure. These switches include a first (middle) position: channel not activated; up position: channel activated; down position: channel activated with 1.5 V offset. A simple 5-led lightning system has been also included in the design to inform about the status of each channel. A reset switch is located at the rear part of the enclosure, to restart the system. Finally, it should be said that one of the advantages of ABDAS design is that the Arduino board can be easily removed.

Besides, ABDAS manufacturing budget is broken down into its different components in Table 3. A total budget of €103.25 in parts has been required in this project. Nevertheless, the budget for this acquisition system can be downgraded to approximately €82, if no offset is required.

**Figure 5:**
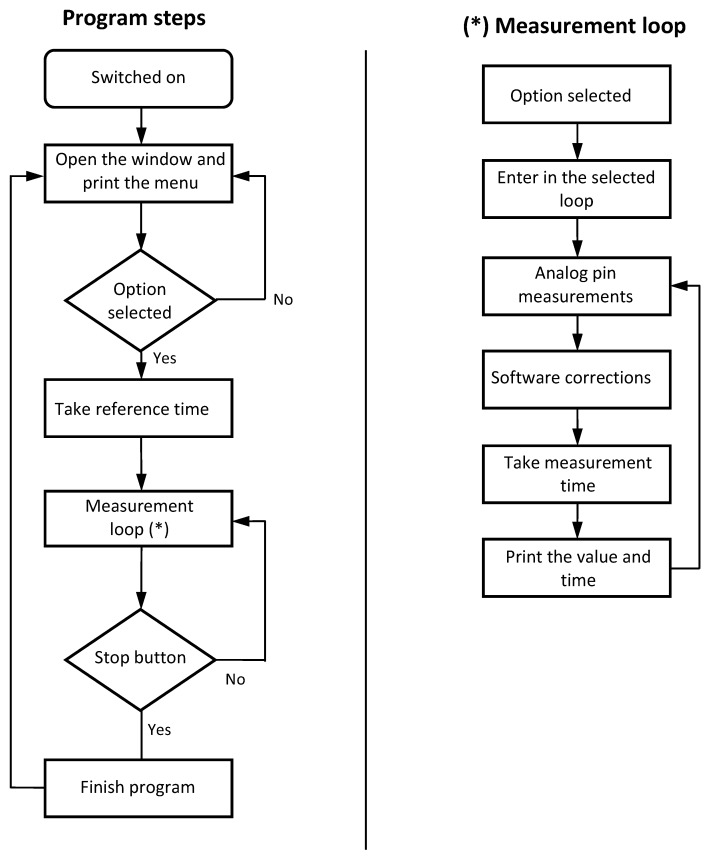
ABDAS software flowchart. Program steps and measurement loop.

**Figure 6:**
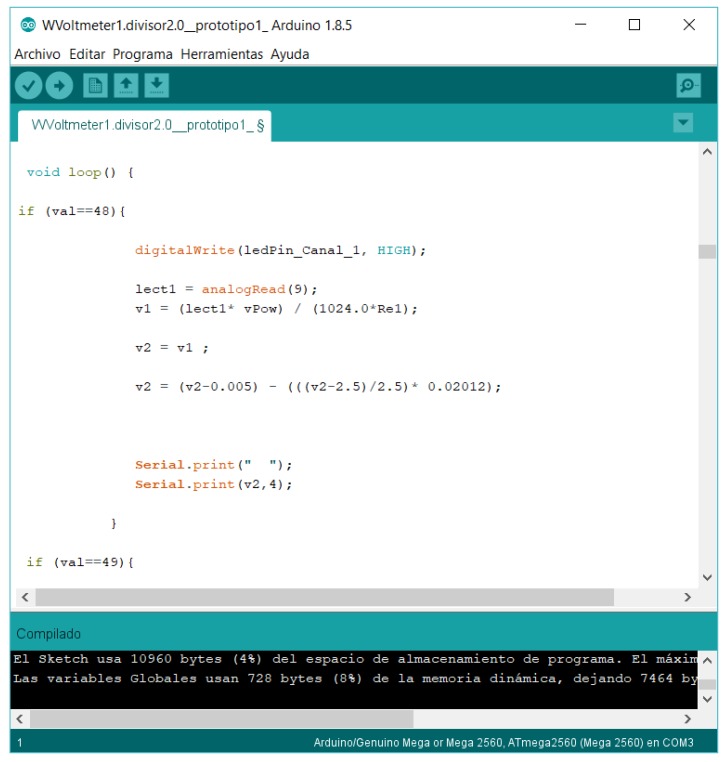
Image capture with part of the ABDAS software control program code.

The software developed for the device must be able to be modified easily to suit future changes in the specific requirements. The code was written using the Arduino language (that is, open source software). The program flow chart is shown in Figure 5. As it can be observed, the measurement loop, which is the algorithm responsible for the data measurements in each channel, is quite short, compact and simple. The results of the voltage dividers’ calibration (Figure 3 and Table 2) are included in the code (“Software correction” in the aforementioned flow chart from Figure 5). See in Figure 6 an image capture of the code program.

**Figure 7:**
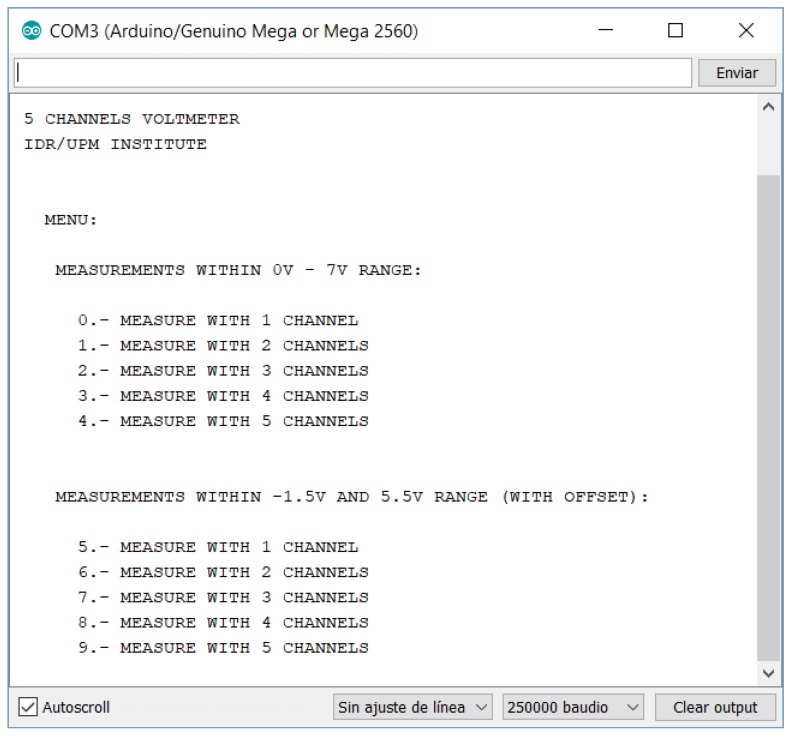
Serial monitor window of ABDAS.

The user interface is the serial monitor window (see Figure 7). In this window, the results are shown. The design has tried to obtain the most easiest and simple interface with the user. Nevertheless, this environment can be easily customized by any potential user.

**Figure 8:**
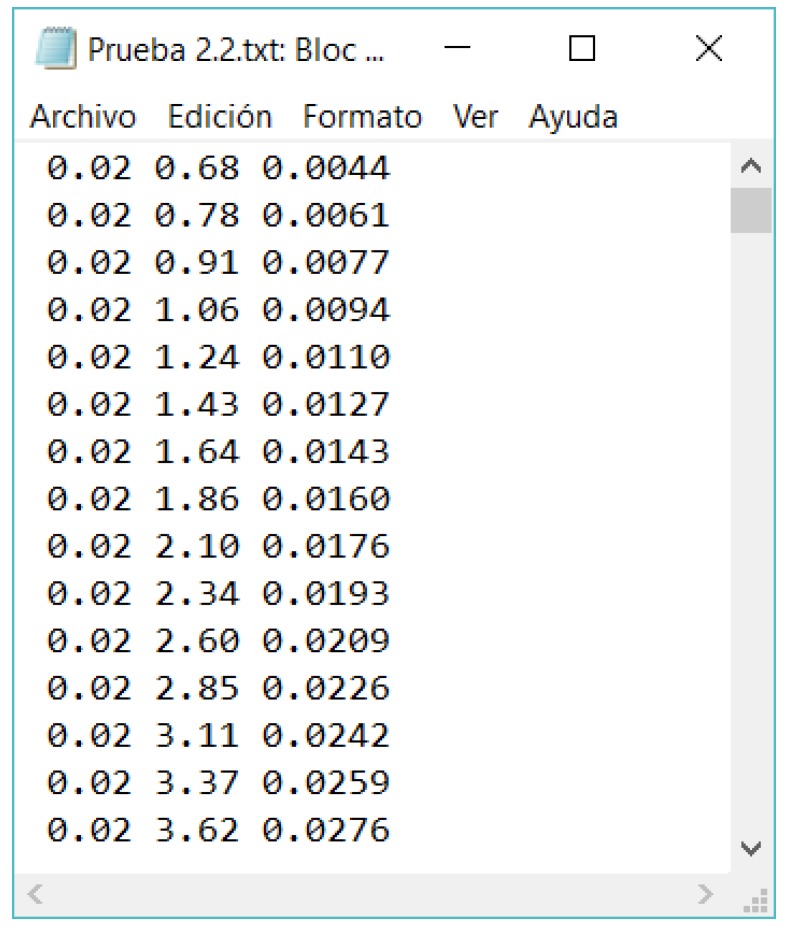
Results from a measurement carried out with ABDAS. Text file example. Two channels measured (two first column). The third column indicates the time of each measurement point (expressed in seconds).

Finally, the results are written in a text file (see Figure 8), corresponding the first columns to the voltage measurement in each channel, whereas the last column corresponds to the time of the measurements taken.

## 3. Experimental Set-Up

**Figure 9:**
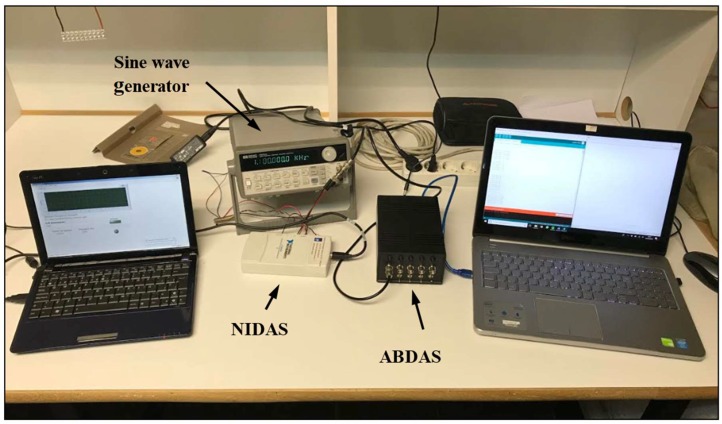
Arduino-based acquisition system (ABDAS) and the National Instruments NI USB-6210 data acquisition system (NIDAS), used to check its performances.

Two different testing campaigns were carried out to test the Arduino-based acquisition system (ABDAS) and analyze its performances. The first one is a direct comparison with reference data obtained with the aforementioned National Instruments NI USB-6210 Data Acquisition System (NIDAS; see Figure 9), whereas in the second testing campaign the output signal of cup anemometers during its calibration was measured with the ABDAS, in order to define the anemometers’ transfer function. This second testing campaign represents a practical example of the use of the developed Arduino-based acquisition system in a well-stablished technical procedure.

**Figure 10:**
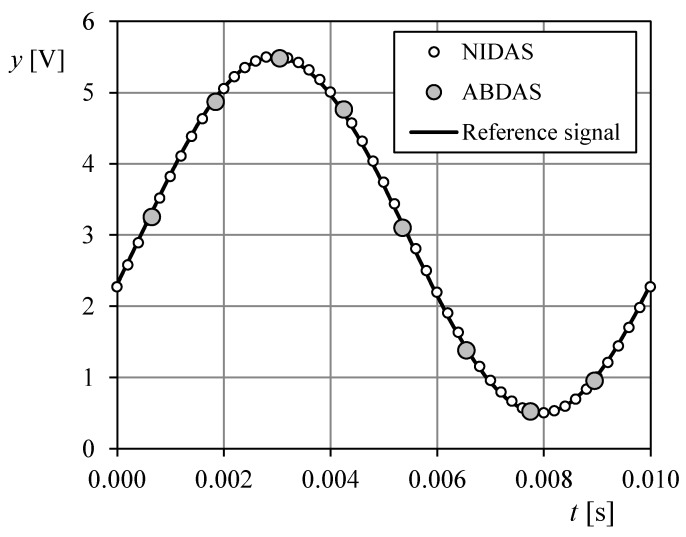
One wave period of the 10 Hz frequency reference signal (Equation (3)). The data measured with NIDAS and ABDAS have been included for comparison purposes.

Within the first testing campaign, both ABDAS and NIDAS measured 10, 50, 100 and 250 Hz sine waves generated by a Hewlett Packard 33120A waveform generator. These signals were characterized by 2.5 V amplitude and 3.0 V offset (see Figure 10). The reference signal is then characterized by the following equation:

(3) y(t)=3+2.5sin(ωt+φ),

where *ω* is the angular frequency (*ω* = 2π*f*; *f* being the frequency of the sine wave), and *ϕ* the phase angle. ABDAS measured at 820 Hz sampling rate, whereas NIDAS was programmed to measure at 5 kHz sampling rate.

Thought ABDAS and NIDAS were not coordinated to start measuring at the same instant, the comparison between both systems was carried out by using the Fourier transform performed on 10 wave periods extracted from each case. As it is well-known, once the Fourier transform coefficients (*y*_0_, *y*_1_, *y*_2_...) have been extracted it is possible to express the measured data as an analytical function:

(4) $$y_{m}\left(t\right)=y_{0}+\sum_{n=1}^{\infty }y_{n}\sin\left(n\omega t+\varphi_{n}\right)$$

Obviously and according to the reference signal (Equation (3)), the data measured by both acquisition systems, ABDAS and NIDAS, should ideally be the same: *y*_0_ = 3; *y*_1_ = 2.5; and *y_i_* = 0 for *i* > 1), leaving aside the possible errors of the sine wave generator.

As previously said, a second testing campaign was scheduled. Along this campaign the output signal from three THIES First Class cup anemometers was sampled during their calibration process, in order to compare the results from ABDAS with the ones from an accredited calibration system.

Cup anemometers, which are the most common wind sensors within meteorology and the wind energy sector, need to be calibrated in order to give the most possible accurate measurement of the wind speed. This calibration represents the definition of the instrument transfer function, which relates the measured wind speed, *V*, to the cup anemometer’s output frequency, *f*:

(5) V=A f+B.

**Figure 11:**
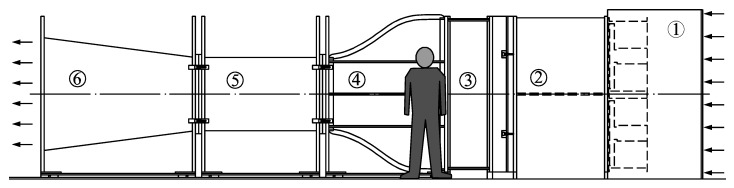
Sketch of the S4 wind tunnel at the IDR/UPM Institute used for anemometer calibration. The different parts of the wind tunnel are indicated in the figure as follows: 1. Fans; 2. Plenum chamber; 3. Honeycomb and grids; 4. Contraction; 5. Test chamber; 6. Diffuser. [11].

In the above Equation, constants A and B are defined by means of a proper calibration. The cup anemometer calibrations used in the present work were performed at IDR/UPM Institute S4 wind tunnel facility (see Figure 11), and follow MEASNET requirements (13 measurement points taken within a wind speed bracket from 4 m·s^−1^ to 16 m·s^−1^, the cup anemometer transfer function (Equation (5) being fitted to these data). The S4 wind tunnel is an open-circuit wind tunnel with a closed test section measuring 0.9 by 0.9 m. It is served by four 7.5 kW fans with a flow uniformity under 0.2% in the testing area. More information on the S4 wind tunnel and the anemometer calibration process followed at IDR/UPM Institute can be found in [11,12,13,22].

## 4. Results and Discussion

### 4.1. First Testing Campaign

The data obtained by ABDAS and NIDAS during one sine wave period (Equation (3)) at 100 Hz frequency are shown in the previously mentioned Figure 6. As it can be observed, the definition of the programmed wave function is much better with the measured data from NIDAS, as expected. The Nyquist theorem implies that number of Fourier harmonic terms that can be properly calculated is limited by the sampling frequency. In Table 4, the number of points per wave, *n_w_*, and the highest harmonic term, *y*_max_, able to be measured in each case by ABDAS and NIDAS are included.

**Figure 12:**
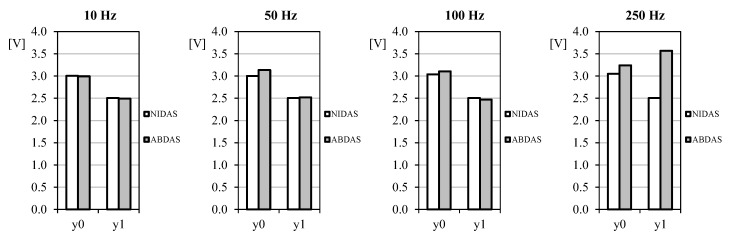
Offset, *y*_0_, and the first harmonic term, *y*_1_, extracted from the sampled data of same signal (Equation (3)) measured with NIDAS and ABDAS.

As said in Section 3, the generated sine-wave signal was measured with both NIDAS and ABDAS during 30 s for each selected frequency. Ten complete wave periods were extracted from all data records, the Fourier series being calculated for all of them. In Figure 12 the averaged values (from the 10 selected wave periods) of the first couple of terms (the offset, *y*_0_, and the first harmonic, *y*_1_) are shown. It can be said that up to 100 Hz frequency signals, measurements carried out with ABDAS are very similar to the ones obtained with NIDAS.

**Figure 13:**
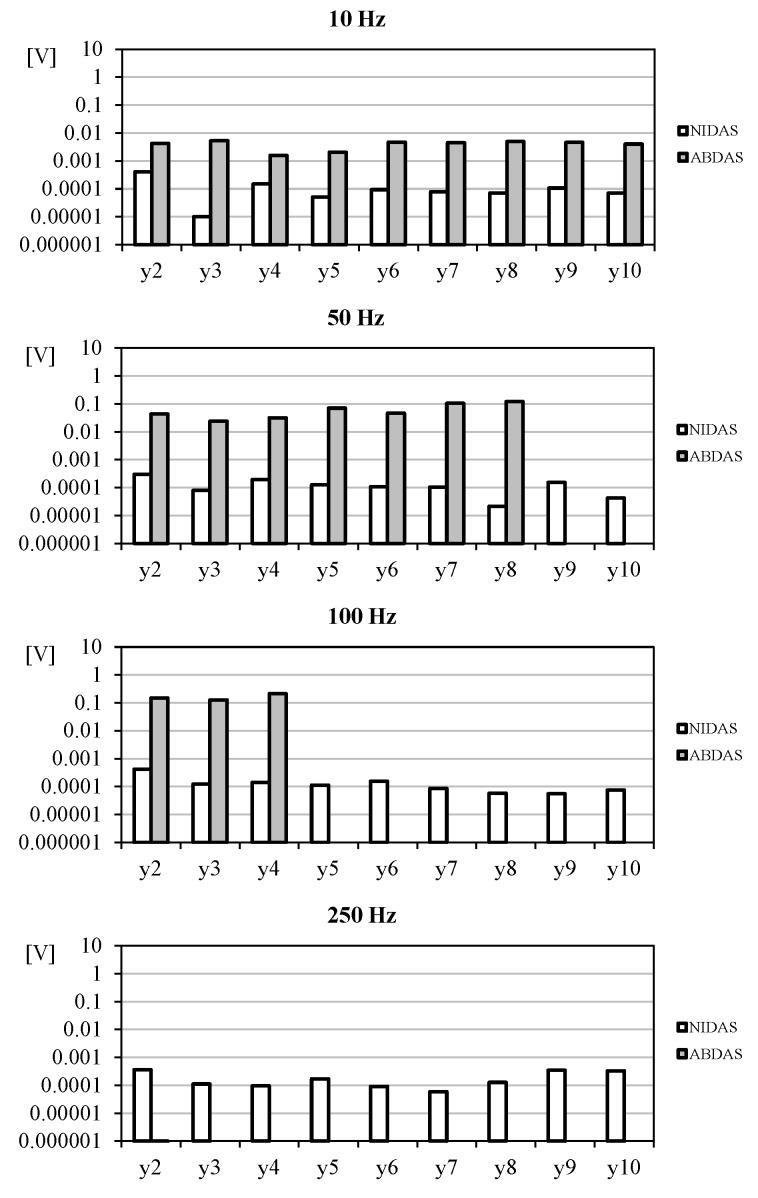
Harmonic terms, *y*_2_ to *y*_10_, extracted from the sampled data of same signal (Equation (3)) measured with NIDAS and ABDAS.

**Figure 14:**
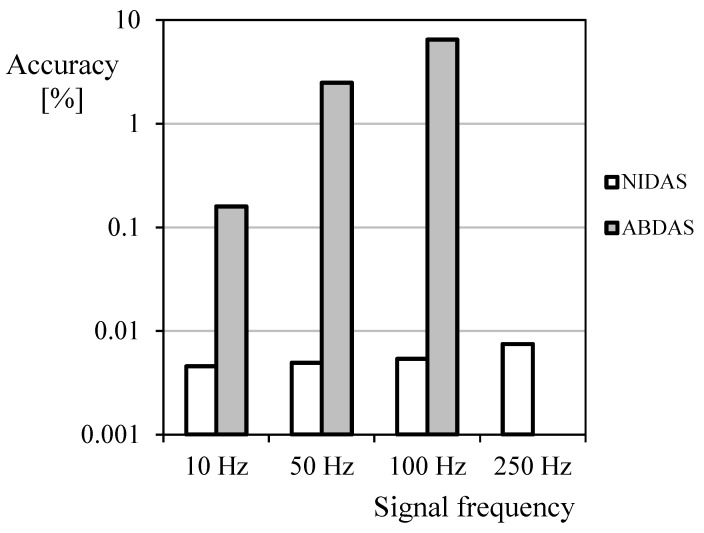
Estimated accuracy ABDAS and NIDAS in relation to the frequency of the signal measured (Equation (3)).

In Figure 13, the correspondent values of the harmonic terms starting from the second one are shown. This information is interesting as it is a way to compare the accuracy (as aforementioned, the second and following terms should be ideally zero). Calculating the mean values from these harmonic terms it is then possible to obtain a rough estimation of the system’s accuracy. As a result, NIDAS seems to have a 0.005–0.008% accuracy in relation to *y*_1_, whereas the accuracy level of ABDAS is around 0.16–6.5% (see Figure 14). Additionally, it can be noted that the bars from ABDAS measurements are missing from the 250 Hz graph (and partially from the 50 Hz and 100 Hz graphs), in accordance with the limitations specified in Table 4. This led us to state that ABDAS is capable of measuring accurately a 50 Hz signal and the related noise up to the eighth harmonic term.

### 4.2. Second Testing Campaign

As said in the previous section, three cup anemometers (hereafter Anemometer-1, Anemometer-2 and Anemometer-3), were used in this testing campaign in order to compare the results measured with ABDAS with an accurate and accredited measuring calibration system (calibration system of LAC-IDR/UPM).

**Figure 15:**
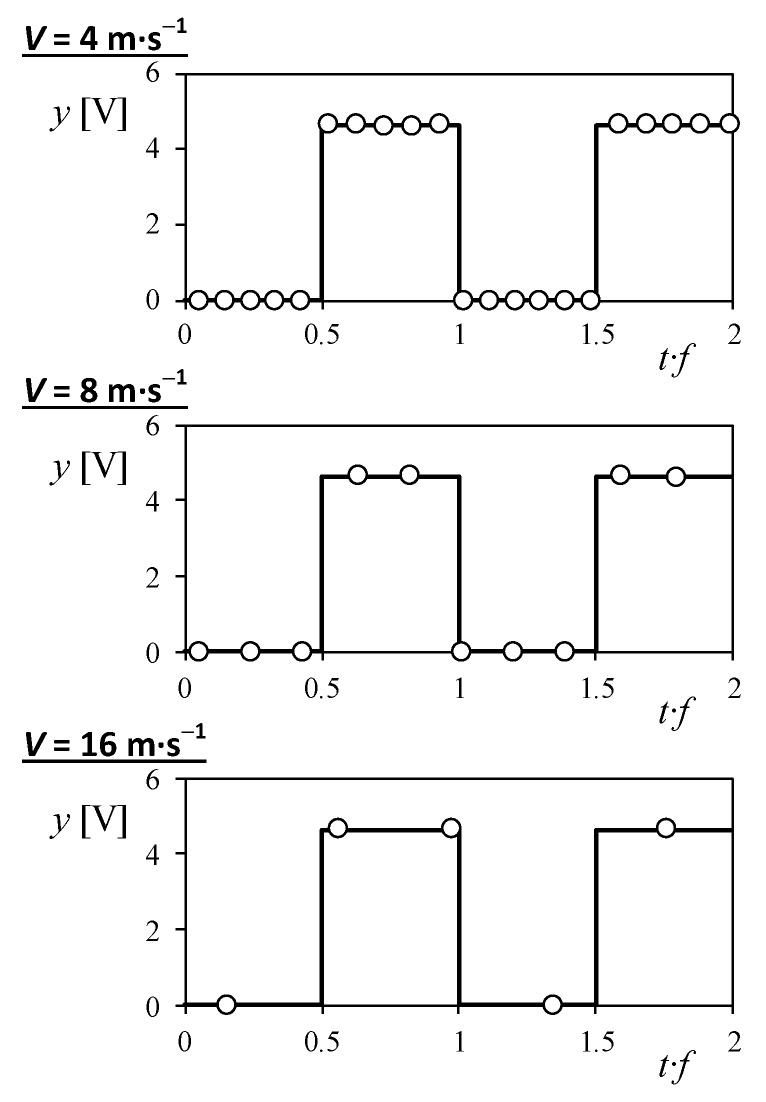
Number sample points (open circles) measured with ABDAS into three 2-period brackets of the three tested cup anemometers’ output signal.

In Figure 15, the sample points obtained by ABDAS along 2 periods of one the three tested cup anemometers’ output signal (square wave), are shown for three different wind velocities, 4, 8, and 16 m·s^−1^. As it is logical, the number of points taken per period decreases with the wind velocities, as these higher wind velocities produce increase the frequency of the output signal. From the data measured with ABDAS from each cup anemometer at all wind velocities, the frequency of the output signal was calculated by two different methodologies used in cup anemometer calibration labs:

- Counting the number of pulses in the data record;
- Calculating numerically the Fourier transform, which indicates the most important frequency from the data record, i.e., the output signal.

The first methodology to obtain the output frequency, *f*, is quite simple, it only requires counting the number of times, *N*, the voltage level rises up to ~4 V from a previous measurement point of ~0 V, within the data recording time, *t_d_*:

(6) $$f=\frac{N}{t_{d}}.$$

On the other hand, the second methodology requires calculating the Fourier transform of the recorded data. In some laboratories, the anemometer’s output frequency is calculated using the Fast Fourier Transform (FFT). In the present cases it was not possible to use such technique, as the time between two consecutive recorded pulses was not equal, that is, there were a certain level of error related to the sampling time step due to the accuracy of ABDAS. Therefore, the direct Fourier transform was used:

(7) $$F\left(f\right)=\frac{1}{\sqrt{2\pi }}\int_{-\infty }^{\infty }y(t)\exp(-i2\pi ft)dt$$

**Figure 16:**
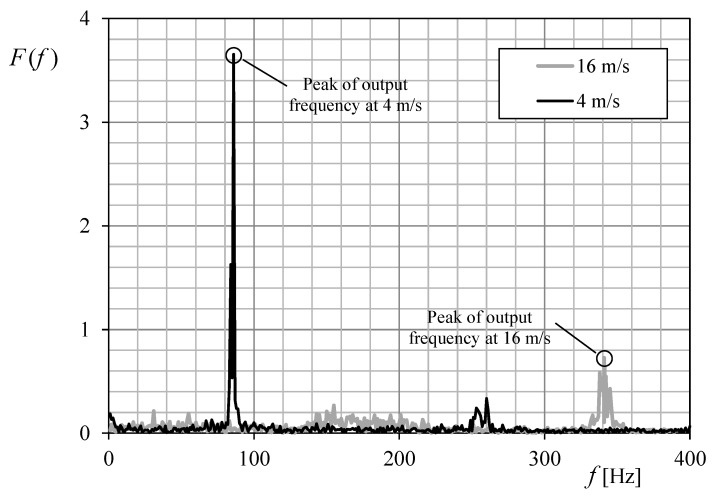
Fourier transforms of the recording data with ABDAS during the calibration process of Anemometer-2, at 4 and 16 m·s^−1^.

**Figure 17:**
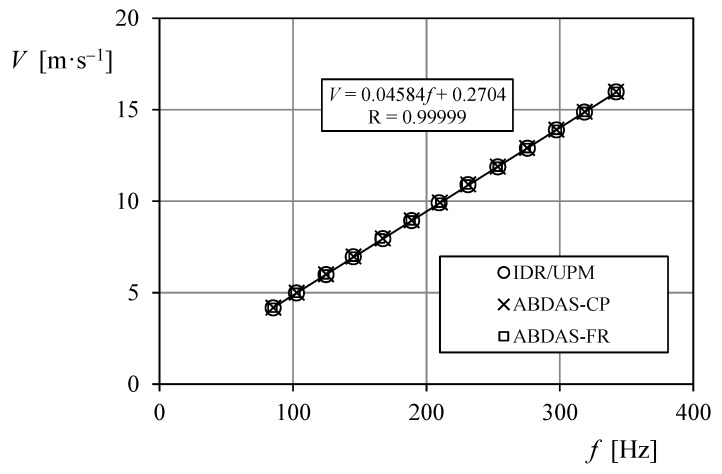
Calibration points of Anemometer-1, obtained from the IDR/UPM calibration system (the transfer function resulting from these data is included in the graph), and from the data measured with ABDAS and post-processed counting pulses (ABDAS-CP; see Equation (6)) and using the Fourier transform (ABDAS-FR; see Equation (7)).

In Figure 16 the Fourier transform of the data recorded from the Anemometer-2 calibration, at 4 and 16 m·s^−1^ is shown. The peak of the transforms reveals the anemometer’s output frequency that, once correlated to the wind speed, defines the anemometer transfer function (see Figure 17). These calculations were carried out by using GNU Octave.

**Figure 18:**
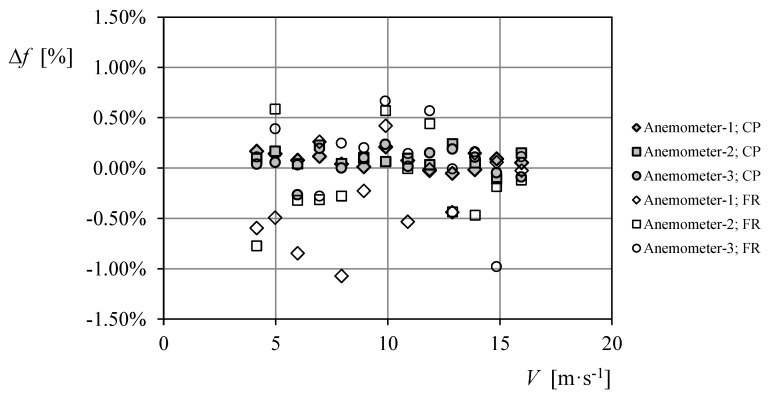
Percentage difference of the frequencies obtained, ∆*f* (Equation (8)), from the ABDAS data measurements using the pulse-counting (CP; Equation (6)) and the Fourier transform (FR; Equation (7)) procedures, related to the ones measured with the IDR/UPM system, *f_IDR_*.

The results of the calibrations performed on the three case study anemometers are included in Table 5. In this table, the calibration coefficients (see Equation (5)) resulting from the accredited calibration system of the IDR/UPM Institute, are compared to the ones from the data recorded with ABDAS, post-processed using both aforementioned procedures, counting pulses (Equation (6)), and using the Fourier transform (Equation (7)). As it can be observed, no big differences seem to be between the calibration constants obtained with the procedures used (see also Figure 17). However, the fitting to the wind speed/frequency data is better correlated to the linear transfer function (Equation (5)) counting pulses than using the Fourier transform, as indicated by the higher values of the correlation coefficient *R*. Bearing in mind that the frequencies obtained with the IDR/UPM accredited calibration system can be assumed to be the most accurate ones, the two procedures for extraction the anemometer output frequency from the data can be compared in relation to that values. In Figure 18, the percentage difference of the frequencies obtained, *f*, in relation to the ones measured with the IDR/UPM system, *f_IDR_*:

(8) $$\Delta f=\frac{f-f_{IDR}}{f_{IDR}}.$$

is shown for the three calibrated anemometers. The graph displayed in this figure indicates a larger deviation of the frequencies based on the Fourier transform from the ones calculated with the reference IDR/UPM calibration system. Furthermore, the standard deviation of these data, *σ*_∆*f*_, is included in Table 5, clearly indicating a poorer results of the calibrations performed using the Fourier transform instead of counting the number of pulses within the recording period.

## 5. Conclusions

A low-cost Arduino-Based Data Acquisition System (ABDAS) for use in the IDR/UPM Institute aerodynamics lab has been designed and developed. This system has proven to be accurate enough to measure signals up to 50 Hz within ±1% error level. Besides, ABDAS has been used to analyze wind sensors (cup anemometers) performance, the most relevant conclusion resulting from this particular case study being:

- ABDAS was able to record data that produce cup anemometer transfer functions similar to the ones obtained with the accredited anemometer calibration system from IDR/UPM;
- The cup anemometer transfer function parameter extraction procedure based on counting pulses, is better than the one based on the Fourier transform in terms of accuracy of the transfer function.

## Figures and Tables

**Table 1:** Technical specifications of Arduino Mega 2560 [70].

Microcontroller	ATmega 2560
Operating Voltage	5 V
Input Voltage (recommended)	7–12 V
Input Voltage (limit)	6–20 V
Digital I/O Pins	54 (of which 15 provide PWM output)
Analog Input Pins	16
DC Current per I/O Pin	20 mA
DC Current for 3.3 V Pin	50 mA
Flash Memory	256 KB (8 KB used by bootloader)
SRAM	8 KB
EEPROM	4 KB
Clock Speed	16 MHz
LED_BUILTIN	13
Length	101.52 mm
Width	53.3 mm

**Table 2:** Transfer function coefficients, *a* and *b*, of the voltage divider installed at each ABDAS input channel. The coefficient of determination, *R*^2^, corresponding to the fitting (see Figure 3) are included, together with the maximum and averaged error, |*ε*|_max_ and |*ε*|_avg_, between the measurements (Figure 3) and the transfer function.

Voltage Divider	*a*	*b* [V]	*R*^2^	|*ε*|_max_ [V]	|*ε*|_avg_ [V]
VD-1	0.66684	−0.00101	1.0	2.5320 × 10^−3^	8.4159 × 10^−4^
VD-2	0.66413	−0.00037	1.0	1.2941 × 10^−3^	6.8456 × 10^−4^
VD-3	0.66810	−0.00066	1.0	1.3404 × 10^−3^	5.3216 × 10^−4^
VD-4	0.66696	−0.00055	1.0	1.5693 × 10^−3^	5.7317 × 10^−4^
VD-5	0.66622	−0.00005	1.0	1.7064 × 10^−3^	6.0411 × 10^−4^

**Table 3:** Characteristics and price of ABDAS components.

Component	Nature	Commercial	Price [€]
Arduino Mega 2560	Obligatory	Yes	42
BNC	Obligatory	Yes	8.65
Switches	Optional	Yes	7.45
Enclosure	Obligatory	Yes	11.11
Resistor	Obligatory	Yes	8.2
Battery holders	Optional	Yes	7.5
Batteries	Optional	Yes	6.2
Cable & welding	Obligatory	No	3.5
Leds	Optional	Yes	7.85
Switching button	Obligatory	Yes	0.79

**Table 4:** Number of points, *n_w_*, measured by NIDAS and ABDAS in one single period of the reference signal, and highest Fourier harmonic term, *y*_max_, able to be correctly extracted.

Reference signal (Sine-Wave) Frequency	NIDAS		ABDAS	
	*n_w_*	*y*_max_	*n_w_*	*y*_max_
10 Hz	500	*y*_249_	83–84	*y*_41_
50 Hz	400	*y*_49_	16–17	*y*_8_
100 Hz	50	*y*_24_	8–9	*y*_4_
250 Hz	20	*y*_9_	3–4	*y*_1_

**Table 5:** Calibration coefficients, A, B, and correlation coefficient *R*, (see Equation (5)) resulting from the calibrations performed to Anemometers 1–3. The coefficients included in the table are the ones obtained from the IDR/UPM accredited calibration process, and the ones from the data measured with ABDAS and post-processed counting pulses (ABDAS-CP; see Equation (6)) and using the Fourier transform (ABDAS-FR; see Equation (7)).

**Anemometer 1**			
**Calibration Constants**	**IDR/UPM Cal. Sys**	**ABDAS-CP**	**ABDAS-FR**
A [m]	0.04584	0.04584	0.04572
B [m·s^−1^]	0.2704	0.2635	0.3128
*R*	0.99999	0.99998	0.99992
*σ*_∆*f*_	-	7.790 × 10^−4^	4.467 × 10^−3^
**Anemometer 2**			
**Calibration Constants**	**IDR/UPM Cal. Sys**	**ABDAS-CP**	**ABDAS-FR**
A [m]	0.04604	0.04602	0.04613
B [m·s^−1^]	0.2449	0.2404	0.2348
*R*	0.99999	0.99999	0.99990
*σ*_∆*f*_	-	9.111 × 10^−4^	4.128 × 10^−3^
**Anemometer 3**			
**Calibration Constants**	**IDR/UPM Cal. Sys**	**ABDAS-CP**	**ABDAS-FR**
A [m]	0.04590	0.04589	0.04602
B [m·s^−1^]	0.2604	0.2568	0.2279
*R*	0.99999	0.99999	0.99987
*σ*_∆*f*_	-	1.366 × 10^−3^	4.068 × 10^−3^
